# Low-Profile Altura^®^ Endograft System Versus Standard-Profile Stent Grafts for Endovascular Aneurysm Repair: A Case-Matched Study

**DOI:** 10.3390/jcm15010293

**Published:** 2025-12-30

**Authors:** Marek Piwowarczyk, Mateusz Rubinkiewicz, Jerzy Krzywoń, Roger M. Krzyżewski, Jeremy Jan Spula, Hubert Kostka, Katarzyna Zbierska-Rubinkiewicz

**Affiliations:** 1 Department of Vascular Surgery, University Hospital of Krakow, 30-688 Krakow, Poland; mpiwowarczyk@su.krakow.pl (M.P.); jkrzywon@gmail.com (J.K.); 2 2nd Department of General Surgery, Jagiellonian University, 31-007 Krakow, Poland; mateusz.rubinkiewicz@uj.edu.pl; 3 Department of Neurosurgery, Jagiellonian University, 31-007 Krakow, Poland; roger.krzyzewski@uj.edu.pl; 4 Faculty of Medicine, Collegium Medicum, Jagiellonian University, 31-008 Kraków, Poland; jeremyspula@gmail.com (J.J.S.); h-kostka99@o2.pl (H.K.)

**Keywords:** endovascular aneurysm repair, abdominal aortic aneurysm, low-profile stent graft

## Abstract

**Background/Objectives:** Endovascular aneurysm repair (EVAR) is currently the preferred method for treating abdominal aortic aneurysms (AAA) due to lower perioperative morbidity and mortality compared with open aortic repair (OAR). However, anatomical limitations such as narrow or tortuous iliac arteries may preclude EVAR. The low-profile Altura^®^ stent graft (LPSG) was designed to overcome these limitations. This study aimed to compare the outcomes of Altura^®^ low-profile endografts with standard-profile stent grafts (SPSGs) in AAA treatment. **Methods:** This single-center, retrospective, case-matched study included 30 patients treated with Altura^®^ LPSG and 30 matched controls who underwent SPSG implantation between July 2021 and February 2023. Demographic, anatomical, operative, and postoperative parameters were analyzed. Follow-up was performed at 3, 6, and 12 months using ultrasound and computed tomography angiography (CTA). **Results:** Patients in the LPSG group more frequently had narrow access vessels (<6 mm, 46.7% vs. 3.3%, *p* = 0.001). The mean procedure time was shorter in the LPSG group (80 vs. 90 min, *p* = 0.04), and hospital stay was reduced (3 vs. 4 days, *p* = 0.03). No 30-day mortality occurred in either group. At 12 months, no aneurysm rupture, graft infection, or aneurysm-related death was observed. The rate of secondary interventions was comparable between groups. **Conclusions:** The low-profile Altura^®^ stent graft provides a safe and effective option for AAA patients with narrow access vessels. Its bilateral parallel configuration and lack of gate cannulation simplify EVAR, shorten procedure time, and may be especially beneficial in emergency or anatomically challenging cases. Further prospective studies are warranted to confirm these findings.

## 1. Introduction

Endovascular aneurysm repair (EVAR) has proved to be superior to open aortic repair (OAR) in early morbidity and mortality in patients with infrarenal abdominal aortic aneurysms (AAAs) [1,2]. Nowadays, more than three-quarters of patients in the United States and some European countries are treated with EVAR for unruptured AAA [3,4]. However, not all patients are eligible for EVAR due to anatomical requirements. EVAR applicability is limited in patients with difficult iliofemoral vascular access, including angulated, tortuous, or narrow iliac arteries. It is a frequent problem, since concomitant iliac artery stenosis is present in 36% of patients with AAA, increasing the risk of injury to the access vessel during the procedure [5]. Fortunately, there are new generations of devices—low-profile stent grafts (LPSGs) with more flexibility and smaller insertion systems’ diameters. They are safer for patients with narrow iliac arteries and enable the procedure to be performed percutaneously and, thus, less invasively. The Altura^®^ endoprosthesis (Lombard Medical, Ltd., Oxford, UK) is a new, low-profile endograft designed to simplify the treatment of infrarenal AAA and optimize both proximal and distal fixation in the setting of uneven and/or problematic anatomy. Two proximal D-shaped self-expanding endografts with suprarenal fixation are used in their innovative design, each in a 14-F delivery system. No gate cannulation is required, which may significantly shorten the procedure time. To maximize possible fixation length, the device’s limbs can be precisely positioned both proximally and distally. The current study compares the short- and mid-term outcomes of standard-profile stent grafts (SPSGs) and Altura^®^ LPSG for EVAR of AAA.

## 2. Materials and Methods

### 2.1. Setting and Design

This retrospective, single-center study with matched cases was conducted in accordance with the principles of the Declaration of Helsinki and approved by the local ethics committee. All participants provided written informed consent before undergoing the surgical procedure. The study was initiated and carried out by the investigators and was not supported by any industry funding.

### 2.2. Patients

From July 2021 to February 2023, 30 patients diagnosed with abdominal aortic aneurysm (AAA) underwent endovascular aortic repair (EVAR) with the use of the Altura^®^ low-profile stent graft. During the same period, 107 patients were treated with standard-profile stent grafts. Hospital admission generally took place one day before the scheduled intervention to allow for appropriate preoperative assessment and preparation. In the absence of postoperative complications, patients were typically discharged on postoperative day one or two, following routine clinical practice and individual medical evaluation.

After data collection, a control cohort consisting of patients treated with standard-profile stent grafts was established using statistical matching performed with MedCalc software (Medcalc 23.3.7). The study included clinical data as well as preoperative and follow-up computed tomography angiography (CTA) from patients who underwent endovascular AAA repair at our institution. Postoperative follow-up visits were scheduled at 3, 6, and 12 months. Surveillance consisted of ultrasound (USG) and CTA to assess aneurysm morphology, stent graft configuration, and vessel patency, which are considered the most reliable imaging modalities for follow-up [4]. Reinterventions were undertaken when clinically indicated.

### 2.3. Operation Details

All patients assigned to the low-profile stent graft (LPSG) group were treated using the Altura^®^ endoprosthesis (Lombard Medical Ltd., Oxford, UK). This device is a self-expanding braided nitinol stent equipped with fixation barbs and radiopaque markers and covered with a seamless, ultra-low-porosity polyester graft material. It is delivered through a low-profile sheath, with a minimum delivery system size of 14 F. A distinctive characteristic of the Altura^®^ system is its bilateral D-shaped stent configuration [6]. The Altura^®^ stent graft was preferentially selected for patients presenting with narrow access vessels, defined as a vessel diameter below 6 mm.

Patients in the standard-profile stent graft (SPSG) group received various conventional devices, including E-tegra (Artivion, Hechingen, Germany) and Endurant (Medtronic Inc., Minneapolis, MN, USA). These stent grafts are composed of a woven polyester fabric attached to a stainless-steel exoskeleton and are introduced via 18–20 F delivery systems. When percutaneous femoral access was employed, vascular closure was achieved using a suture-mediated closure device (ProGlide, Abbott Vascular Devices, Abbott Park, IL, USA), with a single device used for 14 F systems and two devices for larger delivery sheaths.

### 2.4. Measured Outcomes

The primary endpoints included the incidence of type I endoleak, the rate of reintervention, and all-cause mortality at 1 year. Secondary endpoints comprised perioperative complications other than type I endoleak, as well as operative and postoperative measures, including the need for additional intraoperative procedures, blood transfusion requirements, postoperative morbidity, length of hospital stay, and 30-day mortality.

### 2.5. Statistical Analysis

Statistical analyses were conducted using Statistica software, version 13.0 PL (StatSoft Inc., Tulsa, OK, USA). The matching procedure was carried out with MedCalc (MedCalc Software Ltd., Ostend, Belgium). Data with a normal distribution are expressed as mean ± standard deviation, whereas non-normally distributed variables are presented as median with interquartile range (IQR). Categorical variables were compared using Fisher’s exact test. The Shapiro–Wilk test was applied to assess data normality. For quantitative variables following a normal distribution, comparisons were performed using Student’s *t*-test; otherwise, the Mann–Whitney U test was applied. A *p*-value < 0.05 was considered statistically significant. Univariate and multivariate logistic regression analyses were performed to identify potential risk factors.

## 3. Results

Demographic characteristics of the study population are summarized in Table 1. A significantly higher proportion of patients in the low-profile stent graft (LPSG) group presented with small access vessel diameters (<6 mm) compared with the standard-profile stent graft (SPSG) group (46.7% vs. 3.3%, *p* = 0.001), which reflects the preferential use of LPSG in this anatomical setting. In the SPSG cohort, only one patient had limited access vessel narrowing; in this case, preprocedural balloon angioplasty was sufficient before implantation of a standard-profile device. Patients with a narrow distal aorta (<16 mm in diameter) were exclusively treated with LPSG; however, this difference did not reach statistical significance. Apart from these anatomical considerations, no significant differences were observed between the groups regarding baseline demographics, comorbid conditions, aortic morphology, or laboratory parameters.

Regarding perioperative characteristics (Table 2), procedure duration time was significantly higher in the SPSG group (80 min vs. 90 min, *p* = 0.04). Only a few patients in the SPSG group required open access and general anesthesia, but this did not reach statistical significance. There were no other significant differences in perioperative characteristics.

Table 3 presents postoperative characteristics. Interestingly, the length of hospital stay was significantly shorter in the LPSG group (3 vs. 4 days, *p* = 0.03). In both groups, none of the patients died in the 30-day postoperative period. In a 1-year follow-up, there was no rupture, no endograft infection, no conversion to open repair, and no aneurysm-related death (Table 4). Two secondary interventions were necessary in the SPSG group and only one in the LPSG group, which was not a significant difference. The change in aneurysm sac diameter, endoleak, and mortality rate did not differ between the groups. In logistic regression analysis, use of LPSG was not a risk factor for perioperative complications, 1-year reintervention and endoleak, or mortality. Congestive heart failure and coronary artery disease were risk factors for overall mortality. There were no other risk factors of adverse events peri- and postoperatively.

## 4. Discussion

In the era of aging societies, the possibility of minimally invasive treatment of abdominal aortic aneurysms is becoming increasingly important. Due to EVAR, a less invasive procedure, more elderly patients who were previously considered unsuitable for surgery are now being offered AAA repair. With the appearance and spread of low-profile stent grafts, an increasing group of patients with unfavorable aneurysm anatomy, including women and patients with severe atherosclerosis, may qualify for surgical treatment. The importance of low-profile endograft delivery systems is widely acknowledged, given that access-related complications are reported in approximately 5–17% of procedures [7]. One of the most frequent EVAR exclusion criteria, especially for Asians [8] and women [9], is poor access. In the study of Sweet et al., 19% of men and 51% of women had bilateral iliac artery diameters < 6 mm [9]. Beckerman et al. stated that iliac morphology is the main cause (41.6%) for the lack of fulfillment of IFU criteria in off-label EVAR procedures [10]. In our study, 15 patients had an access vessel diameter below 6 mm and would possibly be disqualified from EVAR without a low-profile stent graft. The main reason for conversion to open repair is difficult access through small-caliber, calcified, and diseased iliac arteries, which can result in kinking, limb occlusion, and limb stenosis [7]. Moreover, iliac tortuosity has been linked to limb occlusion and type Ib endoleaks [11]. Owing to its braided nitinol framework, the Altura^®^ endograft offers high flexibility and resistance to radial compression, which may lower the likelihood of limb kinking or occlusion in tortuous or stenotic vessels. In addition, the device allows for optimal iliac fixation through accurate, independent placement of each iliac limb relative to the internal iliac artery, facilitated by its distinctive distal-to-proximal iliac deployment mechanism [12].

The results of this study demonstrate comparable outcomes of LPSG and SPSG, with a slight advantage of LPSG. Clinical trials to date have also proven the safety and efficacy of the Altura^®^ stent graft. A study involving 90 patients reported a technical success rate of 99% and favorable mid-term outcomes, including a 94% clinical success rate at 30 days and 99% at one year. The cumulative major adverse event (MAE) rates were 3% at 6 months and 7% at 1 year. [12]. In another study, the device showed a high technical success rate in both elective and emergent cases, with a 97% clinical success rate and a low reintervention rate at 12 months [13].

In our study, there was a significant difference between the groups in the length of the procedure. The mean duration of the procedure in the LPSG group was 80 min, whereas in the SPSG group, it was 90 min. These are much shorter times than in many previous investigations. A study of Derwich et al. reported a mean time of EVAR procedure 210.7 min [14] and a study of Gupta et al. reported 139 (107–182) min [15]. In a cohort of 284 patients undergoing elective EVAR, the average procedure duration was 200 min. Prolonged operative time was identified as an independent predictor of postoperative adverse outcomes, with the odds increasing by 34% for every additional 30 min of surgery (OR = 1.34, *p* < 0.001). This association extended to both early postoperative mortality (OR = 1.48, *p* = 0.003) and mortality at 6 months of follow-up (OR = 1.28, *p* = 0.025) [16]. On the one hand, longer procedure times are often the result of intraoperative complications, which affect long-term outcomes. On the other hand, extending the procedure time itself is a significant burden on the patient’s organism and should not be ignored. It is possible that, in addition to less invasive access [17,18,19] and anesthesia in some cases, the shortened surgery time resulted in earlier discharge of patients in our study. In the study of Gupta et al. on 11,229 patients who underwent EVAR, prolonged operative time was associated with higher post-discharge morbidity [15]. Moreover, shorter procedure time, especially the time to obtain a seal of the stent graft, may be very important in patients with ruptured AAA. The use of a bilateral parallel endograft configuration streamlines the EVAR procedure by removing the requirement for contralateral gate cannulation. Combined with the benefits of a low-profile 14-F delivery system, this design may be particularly advantageous in the management of ruptured aneurysms [12].

Mid-term data for the low-profile Altura^®^ endograft are still limited but generally encouraging. In our study, we did not have any rupture, no endograft infection, no conversion to open repair, and no aneurysm-related death. Krievins et al. reported early- to mid-term outcomes (median follow-up 12.5 months, up to 4 years) with 99% clinical success at 1 year and no aneurysm-related deaths or ruptures [12]. More recently, Knapsis et al. published a 3-year CT-based follow-up of 40 patients treated with the ALTURA^®^™ device, showing significant graft shortening and distal sealing-zone reduction, but again no type I endoleaks, aneurysm ruptures, or AAA-related deaths in mid-term follow-up [20]. Additionally, a single-center series of 100 patients has reported encouraging 5-year results, although only in abstract form [21].

There are other low-profile stent graft systems worth mentioning. We have recently published a case-matched study comparing outcomes of endovascular abdominal aortic aneurysm repair (EVAR) using low-profile stent grafts (LPSGs) versus standard-profile stent grafts (SPSGs) [22]. We showed no aneurysm ruptures, no infections, no conversions to open repair, and no aneurysm-related deaths in either group at 1-year follow-up. However, direct head-to-head comparative studies between the low-profile INCRAFT and ALTURA^®^ stent graft systems are currently lacking. The available literature consists mainly of single-center case series, clinical experience reports for each device separately, and reviews assessing their overall anatomical applicability. As a result, any comparison of INCRAFT and ALTURA^®^ must be made indirectly, based on data derived from separate studies. This represents an important limitation of the current evidence and underscores the need for future prospective, comparative research.

It is important to acknowledge the limitations of our study. The study’s design is retrospective. The number of patients is also limited, which clearly leads to selection bias. In order to reduce selection bias, we decided to design a case-matched study. A randomized study would definitely be more reliable. We also did not collect detailed data about aortic neck length, angulation, and diameter. However, all patients had necks longer than 10 mm, and the angulation was below 60 degrees. If the neck is below 10 mm, we submit patients to fenestrated grafts. Moreover, for neck angulation above 60 degrees, only stents produced by Gore^®^ have proper certification; therefore, we did not submit those patients for Altura^®^ stent grafts. Moreover, only one patient in the standard-profile stent graft had narrow access to the vessels. Nevertheless, 50 percent of patients treated with low-profile stents had normal vessel access. Thus, we think that patients with normal access could also benefit from the low-profile technique. However, that may be a source of bias.

## 5. Conclusions

Our study shows that the use of low-profile Altura^®^ stent grafts is a safe and viable method for patients, especially with narrow access vessels. Moreover, its bilateral parallel endograft system without the need for gate cannulation shortens procedure time and might be important, especially in ruptured AAA. More studies, preferably randomized controlled trials, are needed to confirm the usefulness and safety of the Altura^®^ stent graft.

## Figures and Tables

**Table 1:** Baseline patient characteristics.

Variables	All (*n* = 60)	LPSG (*n* = 30)	SPSG (*n* = 30)	*p*-Value
Age, years	74 (56–88)	73.5 (61–87)	74.5 (56–88)	0.5
Male	50 (83.3%)	26 (86.7%)	24 (80%)	0.73
History of				
Congestive heart failure	21 (35%)	13 (43%)	8 (26.7%)	0.19
Coronary artery disease	18 (30%)	9 (30%)	9 (30%)	1
PTCA/CABG	14 (23.3%)	8 (26.7%)	6 (20%)	0.76
Chronic obstructive pulmonary disease	5 (8.3%)	2 (6.7%)	3 (10%)	1
Atrial fibrillation	7 (11.7%)	4 (13.3%)	3 (10%)	1
Chronic kidney disease	14 (24.1%)	7 (23.3%)	7 (23.3%)	1
Diabetes mellitus type 2	21 (35%)	11 (36.7%)	10 (33.3%)	1
Arterial hypertension	56 (93.3%)	28 (93.3%)	28 (93.3%)	1
Dyslipidemia	41 (68.3%)	21 (70%)	20 (66.7%)	1
Smoking	27 (45%)	15 (50%)	12 (40%)	0.6
Aortic morphology				
Maximum aortic diameter (mm)	54 (37–90)	54 (40–90)	51 (37–77)	0.4
Challenging distal aorta	3 (5%)	3 (10%)	0 (0%)	0.24
Narrow access vessels < 6 mm luminal diameter	15 (25%)	14 (46.7%)	1 (3.3%)	0.001
Laboratory test				
White blood cell count	7.72 (4.91–12.81)	7.55 (5–11.42)	8.05 (4.91–12.81)	0.45
Red blood cell count	4.61 (3.37–5.53)	4.69 (3.37–5.53)	4.64 (3.85–5.5)	0.62
Hemoglobin (g/dL)	14.2 (10.2–17.7)	14.3 (10.2–17.7)	14 (10.9–17.4)	0.72
Platelet count	194 (137–391)	194 (144–391)	197 (137–355)	0.75
Creatinine level	82.3 (50.2–330)	89.2 (50.2–330)	80 (50–130)	0.06
GFR	81 (17–90)	79.5 (17–90)	81.5 (50–90)	0.16

PTCA—Percutaneous transluminal coronary angioplasty, CABG—coronary artery bypass graft, GFR—glomerular filtration rate.

**Table 2:** Perioperative characteristics.

Variables	All (*n* = 60)	LPSG (*n* = 30)	SPSG (*n* = 30)	*p*-Value
Vascular access				
Percutaneous	48 (80%)	25 (83%)	23 (77%)	0.85
Open	5 (8%)	0	5 (17%)	
Hybrid	7 (12%)	5 (17%)	2 (6%)	
Anesthesia				
Local	51 (85%)	28 (94%)	23 (77%)	0.035
Regional	5 (8%)	2 (6%)	3 (10%)	
General	4 (7%)	0	4 (13%)	
Procedure time (min)	90 (55–270)	80 (55–125)	90 (65–270)	0.04
Perioperative complications	4 (6.7%)	1 (3.3%)	3 (10%)	0.61
Additional intraoperative procedures	4 (6.7%)	1 (3.3%)	3 (10%)	0.61
Intraoperative endoleak	3 (5%)	1 (3%)	2 (6.7%)	1
Blood transfusion	2 (3.3%)	0 (0%)	2 (6.7%)	0.49
30-day mortality	0	0	0	-

**Table 3:** Postoperative characteristics.

Variables	All (*n* = 60)	LPSG (*n* = 30)	SPSG (*n* = 30)	*p*-Value
Length of hospital stay	3 (3–10)	3 (3–10)	4 (3–6)	0.03
White blood cell count	9.13 (2.23–19.67)	9.26 (2.23–15.27)	9.06 (5.05–19.67)	0.98
Red blood cell count	3.93 (2.76–5.4)	3.94 (2.76–5.4)	3.97 (2.85–5.01)	0.73
Hemoglobin level (g/dL)	12.45 (7.1–16.5)	12 (8.4–16.5)	12.6 (7.1–14.5)	0.96
Platelet count	167 (72–382)	170 (72–382)	158 (89–281)	0.55
Creatinine level	84.9 (50.5–341)	86.85 (58.3–341)	82 (50–143)	0.26
GFR	81 (17–90)	79.5 (17–90)	84 (45–90)	0.26
Hemoglobin level decrease (g/dL)	1.8 (−0.1–5.5)	1.8 (0.1–5.5)	2.1 (−0.1–5.9)	0.59
Creatinine level increase	−1.1 (−61–253)	−3 (−61–253)	1.5 (−24–46.8)	0.15
GFR level decrease	0 (−25–63)	1.5 (−22–63)	0 (−25–35)	0.34

**Table 4:** Follow-up characteristics.

Variables	All (*n* = 60)	LPSG (*n* = 30)	SPSG (*n* = 30)	*p*-Value
Change in aneurysm sac diameter	−6 (−19–14)	−7 (−19–0)	−6 (−15–14)	0.19
Endoleak	5 (8.3%)	3 (10%)	2 (6.7%)	1
Aortic rupture	0	0	0	-
Endograft infection	0	0	0	-
Conversion to OR	0	0	0	-
Reintervention	3 (5%)	2 (6.7%)	1 (3.3%)	1
Mortality	3 (5%)	1 (3.3%)	2 (6.7%)	1

## Data Availability

The clinical data will be available on individual request to the corresponding author.

## Abbreviations

The following abbreviations are used in this manuscript:

AAA	Abdominal Aortic Aneurysm
EVAR	Endovascular Aneurysm Repair
OAR	Open Aortic Repair
LPSG	Low-Profile Stent Graft
SPSG	Standard-Profile Stent Graft
USG	Ultrasonography
CTA	Computed Tomography Angiography
SD	Standard Deviation
IQR	Interquartile Range
MAE	Major Adverse Events
OR	Odds Ratio
